# Risk factors for short-term mortality in elderly hip fracture patients with complicated heart failure in the ICU: A MIMIC-IV database analysis using nomogram

**DOI:** 10.1186/s13018-023-04258-7

**Published:** 2023-11-03

**Authors:** Yining Lu, Wei Chen, Yuhui Guo, Yujing Wang, Ling Wang, Yingze Zhang

**Affiliations:** 1 Department of Orthopedic Research Center, The Third Hospital of Hebei Medical University, Shijiazhuang, Hebei People’s Republic of China; 2 Department of Orthopedic Surgery, The Third Hospital of Hebei Medical University, Shijiazhuang, Hebei People’s Republic of China; 3 Department of Orthopedic Oncology, The Third Hospital of Hebei Medical University, Shijiazhuang, Hebei People’s Republic of China

**Keywords:** 30-day all-cause mortality, Geriatric hip fractures, Heart failure, Nomogram, Infection, Neutrophils

## Abstract

**Background:**

Hip fracture is a prevalent and hazardous injury among the elderly population that often results in intensive care unit (ICU) admission due to various complications, despite advanced medical science. One common complication experienced in the ICU by elderly hip fracture patients is heart failure, which significantly impacts short-term survival rates. Currently, there is a deficit of adequate predictive models to forecast the short-term risk of death following heart failure for elderly hip fracture patients in the ICU. This study aims to identify independent risk factors for all-cause mortality within 30 days for elderly patients with hip fractures and heart failure while in the ICU in order to develop a predictive model.

**Method:**

A total of 641 elderly patients with hip fractures combined with heart failure were recruited from the Medical Information Mart for Intensive Care IV dataset and randomized to the training and validation sets. The primary outcome was all-cause mortality within 30 days. The least absolute shrinkage and selection operator regression was used to reduce data dimensionality and select features. Multivariate logistic regression was used to build predictive models. Consistency index (C-index), receiver operating characteristic curve, and decision curve analysis (DCA) were used to measure the predictive performance of the nomogram.

**Result:**

Our results showed that these variables including MCH, MCV, INR, monocyte percentage, neutrophils percentage, creatinine, and combined sepsis were independent factors for death within 30 days in elderly patients with hip fracture combined with heart failure in the ICU. The C-index was 0.869 (95% CI 0.823–0.916) and 0.824 (95% CI 0.749–0.900) for the training and validation sets, respectively. The results of the area under the curve and decision curve analysis (DCA) confirmed that the nomogram performed well in predicting elderly patients with hip fractures combined with heart failure in the ICU.

**Conclusion:**

We developed a new nomogram model for predicting 30-day all-cause mortality in elderly patients with hip fractures combined with heart failure in the ICU, which could be a valid and useful clinical tool for clinicians for targeted treatment and prognosis prediction.

## Introduction

With the accelerated aging process, medical care for elderly patients has become a major concern regarding people's quality of life. Among this population, the high incidence of fracture diseases has imposed a large medical burden on both patients and the healthcare system [1]. In particular, hip fracture is one of the most common types of fractures in elderly patients, and their incidence increases with age [2]. Hip fractures can be classified into various types, such as femoral neck fractures and intertrochanteric fractures, depending on the location, direction, and type of fracture [3]. The number of elderly patients worldwide suffering from hip fractures was reported to be 1.3 million in 1990, which is expected to reach 7–21 million by 2050 based on long-term trends [4]. Due to the high morbidity and mortality associated with conservative management, most hip fracture cases are usually managed surgically unless there are contraindications to surgery [3]. However, patients undergoing surgery may face considerable perioperative and postoperative complications including cardiac arrhythmias, pulmonary embolism, or pulmonary infections [5]. Approximately one-quarter of surgical patients will experience adverse cardiac events [6]. Despite the efforts of multidisciplinary collaborative teams, some patients still require further treatment in the intensive care unit (ICU) [7], which significantly drains medical resources. Hip fractures are a significant cause of injury death in the elderly [8, with studies showing that mortality in the first year after a fracture is five times higher in men and three times higher in women compared to the general population [9]. The situation for patients in the ICU is undoubtedly more aggressive; thus, it is clinically important to identify high-risk patients upon admission to the ICU.

Heart failure is a common complication in patients with hip fractures in the ICU and is associated with various factors such as loss of mobility, decreased cardiopulmonary function, and specific therapeutic procedures in the ICU, such as anesthetics, analgesics, antibiotics, and other drugs, which can severely affect cardiac function and aggravate the condition of patients with heart failure [10]. Heart failure also predisposes to many other serious complications such as myocardial infarction, stroke, and delirium [11, 12]. These diseases increase the risk of death in patients. Heart failure greatly affects the patient's cardiac function and predisposes them to cardiac accidents. Additionally, patients with combined heart failure have reduced physical activity, longer postoperative recovery times, and higher rates of deterioration, which may worsen the condition of hip fracture patients. Hip fracture patients are already a high-risk population, and their health status has further deteriorated with combined heart failure. The higher surgical risk of hip fracture, the significant risk of perioperative and postoperative bleeding and infection, and the frequent need for long-term antimicrobial therapy all contribute significantly to patient mortality [13]. The severe nature of the comorbidities in these patients requires more thorough medical management. In some areas where medical resources are relatively limited, comorbidities may be treated less effectively, resulting in higher mortality rates.

To address this issue, we extracted data from patients based on the MIMIC-IV database. We conducted a study to investigate the risk factors associated with 30-day mortality in patients with hip fractures and comorbid heart failure disease who were treated in ICU wards. Our findings allowed us to establish a Nomogram with predictive effect, which can be helpful for clinical management and prognostic assessment. Our research provides new insights for future clinical treatments.

## Materials and methods

### Data sources

Our data were obtained from the MIMIC-IV database, a database created and open-sourced by the Massachusetts Institute of Technology (Massachusetts, USA), which contains information on more than 58,000 patients who have been seen at Beth Israel Deaconess Medical Center. We completed an online course offered by the National Institutes of Health (NIH) and were granted access to the MIMIC-IV database (certification number: 10956161). Data were extracted from the MIMIC-IV database using Structured Query Language (SQL) and pgAdmin4 PostgreSQL 9.6.

### Study population

Information related to each patient's unique HADM_ID was extracted from the MIMIC-IV database using the Structured Query Language (SQL) of PostgreSQL (University of California, Berkeley, version 9.6). With the International Classification of Diseases, 9th edition (ICD-9), we obtained 4072 patients. For multiple admissions, we kept information only for the first ICU admission of the patient. Finally, 641 patients were included in the study.

### Clinical variables and definitions

As described above, using the patients' HADM_ID and ICUSTAY_ID, we extracted the following data: demographics, laboratory tests, comorbidities, and scoring system. Among them, demographic data included age, gender, race, number of fractures; laboratory tests included hematocrit, hemoglobin, mean red blood cell hemoglobin volume (MCH), mean red blood cell hemoglobin concentration (MCHC), mean red blood cell volume (MCV), platelet, red blood cells (RBC), red blood cell distribution width (RDW), international normalized ratio (inr), prothrombin time (pt), partial thromboplastin time (ptt), basophils, eosinophils, lymphocytes percentage, monocytes percentage, neutrophils percentage, anion gap, bicarbonate, urea nitrogen (BUN), chloride, creatinine, glucose, sodium, and potassium; comorbidities included deep vein thrombosis, pulmonary embolism sepsis, peripheral vascular disease, cerebrovascular disease, dementia, chronic lung disease, rheumatic disease, peptic ulcer disease, paraplegia renal disease, malignancy, severe liver disease, metastatic solid tumors, and myocardial infarction; scoring system included Charlson Comorbidity Index. Indicators with > 10% missing data, such as height, weight, blood pressure, heart rate, etc., were removed. All of the above variables were collected for the first time after the patient was admitted to the ICU.

### Statistical analysis

Statistical analysis was performed using the R software (Version 4.1.3; R Foundation for Statistical Computing, Vienna, Austria; https://www.r-project.org). The distribution of continuous variables was assessed using the Lillefors test (based on the Kolmogorov–Smirnov test). Mean and standard deviation (SD) are reported for continuous variables having a normal distribution; median and interquartile range (IQR), for continuous variables having a skewed distribution; and counts and percentages, for categorical variables. Multiple imputations using chained equations were adopted to replace the missing values. The least absolute shrinkage and selection operator (LASSO) method, which accounts for multicollinearity and avoids model overfit, was used to select the optimal candidate variable for constructing the nomogram. Sensitivity and specificity were used to evaluate the model’s performance. The calibration C-index (bootstrap resampling performed 1000 times), the calibration curve, and receiver operating characteristic (ROC) curves were used to evaluate the discrimination and calibration of the model. Decision curve analysis (DCA) was used to evaluate the clinical usefulness of the model. All significance tests were two-sided, and a *P* value < 0.05 was considered statistically significant.

## Result

### Clinical characteristics

**Fig. 1:**
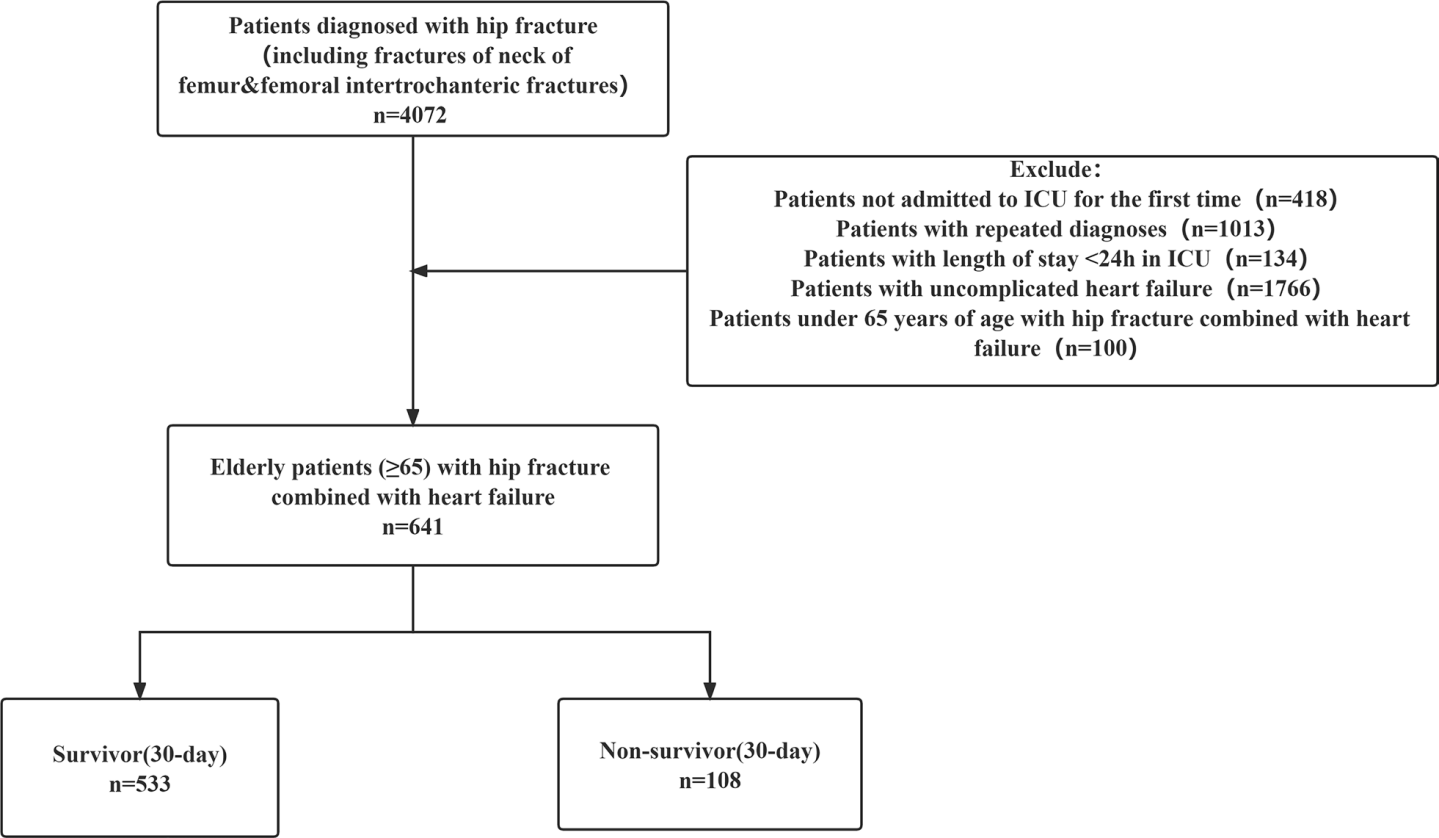
The flowchart of patient selection

A total of 4072 patients were initially included, and 3431 were excluded according to exclusion criteria (Fig. 1). Finally, 641 eligible patients with a mean age of 82.6 years were analyzed (Table 1), 62.4% (*n* = 400) were female with a mortality rate of 18.7%, and 37.6% (*n* = 241) were male patients with a mortality rate of 23.0%. Table 1 compares the differences in characteristics between the inpatient death and survival groups. Compared with the survival group, the death group had a greater proportion of patients who were male and a greater proportion of patients with both combined fractures. Also, hemoglobin and MCV were lower in the death group, while INR, PT, and PTT values were relatively high, indicating poorer coagulation, and BUN and creatinine were higher, indicating poorer renal metabolic function in the death group. In addition, patients in the death group had a high percentage of neutrophils and a much higher percentage of combined sepsis than in the non-death group, suggesting that inflammation and infection may be important factors influencing mortality in patients with hip combined heart failure in the ICU.

**Table 1:** Demographic characteristics of elderly patients with hip fracture combined with heart failure

Characteristics	Survive	Death	*p*. overall
	*N* = 533	*N* = 108	
**Age**	84.0 [78.0, 89.0]	83.0 [78.0, 88.0]	0.344
**Ethnicity**			0.121
Other	66 (12.4%)	20 (18.5%)	
White	467 (87.6%)	88 (81.5%)	
**Gender**			0.396
Female	337 (63.2%)	63 (58.3%)	
Male	196 (36.8%)	45 (41.7%)	
**Number of comorbid fractures**			0.087
Suffering from one type of fracture	378 (70.9%)	67 (62.0%)	–
Suffering from two type of fracture	155 (29.1%)	41 (38.0%)	–
**Hematocrit**	31.5 [27.5, 35.8]	30.3 [27.4, 35.2]	0.173
**Hemoglobin**	10.2 [8.90, 11.7]	9.55 [8.50, 11.5]	0.037
**MCH**	30.3 [28.9, 31.6]	28.0 [26.1, 29.9]	< 0.001
**MCHC**	32.6 [31.7, 33.5]	32.3 [31.1, 33.5]	0.027
**MCV**	92.0 [88.0, 97.0]	86.0 [81.8, 91.0]	< 0.001
**Platelet**	214 [158, 286]	226 [156, 314]	0.302
**RBC**	3.44 [2.97, 3.95]	3.53 [3.12, 4.04]	0.108
**RDW**	14.9 [13.9, 16.3]	15.5 [14.4, 17.0]	0.007
**inr**	1.20 [1.10, 1.50]	1.50 [1.20, 2.10]	< 0.001
**pt**	13.5 [12.1, 16.8]	16.2 [13.8, 23.1]	< 0.001
**ptt**	32.1 [28.1, 36.2]	37.7 [31.9, 43.6]	< 0.001
**Basophils**	0.30 [0.20, 0.60]	0.20 [0.10, 0.40]	< 0.001
**Eosinophils**	1.20 [0.30, 2.60]	0.50 [0.20, 1.10]	< 0.001
**Lymphocytes percentage**	13.8 [8.30, 21.1]	6.95 [4.50, 10.8]	< 0.001
**Monocytes percentage**	5.70 [4.00, 7.80]	3.60 [2.80, 5.30]	< 0.001
**Neutrophils percentage**	76.2 [68.1, 84.5]	87.8 [84.0, 90.7]	< 0.001
**anion gap**	14.0 [12.0, 16.0]	14.0 [12.0, 17.0]	0.143
**bicarbonate**	26.0 [23.0, 28.0]	26.0 [23.0, 29.0]	0.731
**BUN**	24.0 [16.0, 35.0]	31.0 [20.0, 49.8]	0.001
**Chloride**	102 [99.0, 106]	102 [98.8, 106]	0.97
**Creatinine**	1.00 [0.80, 1.50]	1.30 [0.80, 2.22]	0.001
**Glucose**	125 [99.0, 141]	124 [92.8, 141]	0.25
**Sodium**	139 [136, 141]	138 [136, 142]	0.582
**Potassium**	4.20 [3.80, 4.50]	4.25 [3.80, 4.62]	0.451
**DVT**			0.716
No	506 (94.9%)	101 (93.5%)	–
Yes	27 (5.07%)	7 (6.48%)	–
**Pulmonary embolism**			0.373
No	495 (92.9%)	97 (89.8%)	–
Yes	38 (7.13%)	11 (10.2%)	–
**Sepsis**			< 0.001
No	422 (79.2%)	58 (53.7%)	–
Yes	111 (20.8%)	50 (46.3%)	–
**Peripheral vascular disease**			0.57
No	463 (86.9%)	91 (84.3%)	–
Yes	70 (13.1%)	17 (15.7%)	–
**Cerebrovascular disease**			0.306
No	484 (90.8%)	94 (87.0%)	–
Yes	49 (9.19%)	14 (13.0%)	–
**Dementia**			1
No	468 (87.8%)	95 (88.0%)	–
Yes	65 (12.2%)	13 (12.0%)	–
**Chronic pulmonary disease**			0.565
No	378 (70.9%)	73 (67.6%)	–
Yes	155 (29.1%)	35 (32.4%)	–
**Rheumatic disease**			1
No	503 (94.4%)	102 (94.4%)	
Yes	30 (5.63%)	6 (5.56%)	
**Peptic ulcer disease**			0.679
No	525 (98.5%)	106 (98.1%)	–
Yes	8 (1.50%)	2 (1.85%)	–
**Paraplegia**			1
No	526 (98.7%)	107 (99.1%)	–
Yes	7 (1.31%)	1 (0.93%)	–
**Renal disease**			0.101
No	353 (66.2%)	62 (57.4%)	–
Yes	180 (33.8%)	46 (42.6%)	–
**Malignant cancer**			0.097
No	477 (89.5%)	90 (83.3%)	–
Yes	56 (10.5%)	18 (16.7%)	–
**Severe liver disease**			0.628
No	527 (98.9%)	106 (98.1%)	–
Yes	6 (1.13%)	2 (1.85%)	–
**Metastatic solid tumor**			1
No	509 (95.5%)	104 (96.3%)	–
Yes	24 (4.50%)	4 (3.70%)	–
**Myocardial infarct**			0.394
No	450 (84.4%)	87 (80.6%)	–
Yes	83 (15.6%)	21 (19.4%)	–
**Charlson Comorbidity Index**	7.00 [5.00, 8.00]	7.00 [6.00, 9.00]	0.015

### Characteristics selection and development of a nomogram

**Fig. 2:**
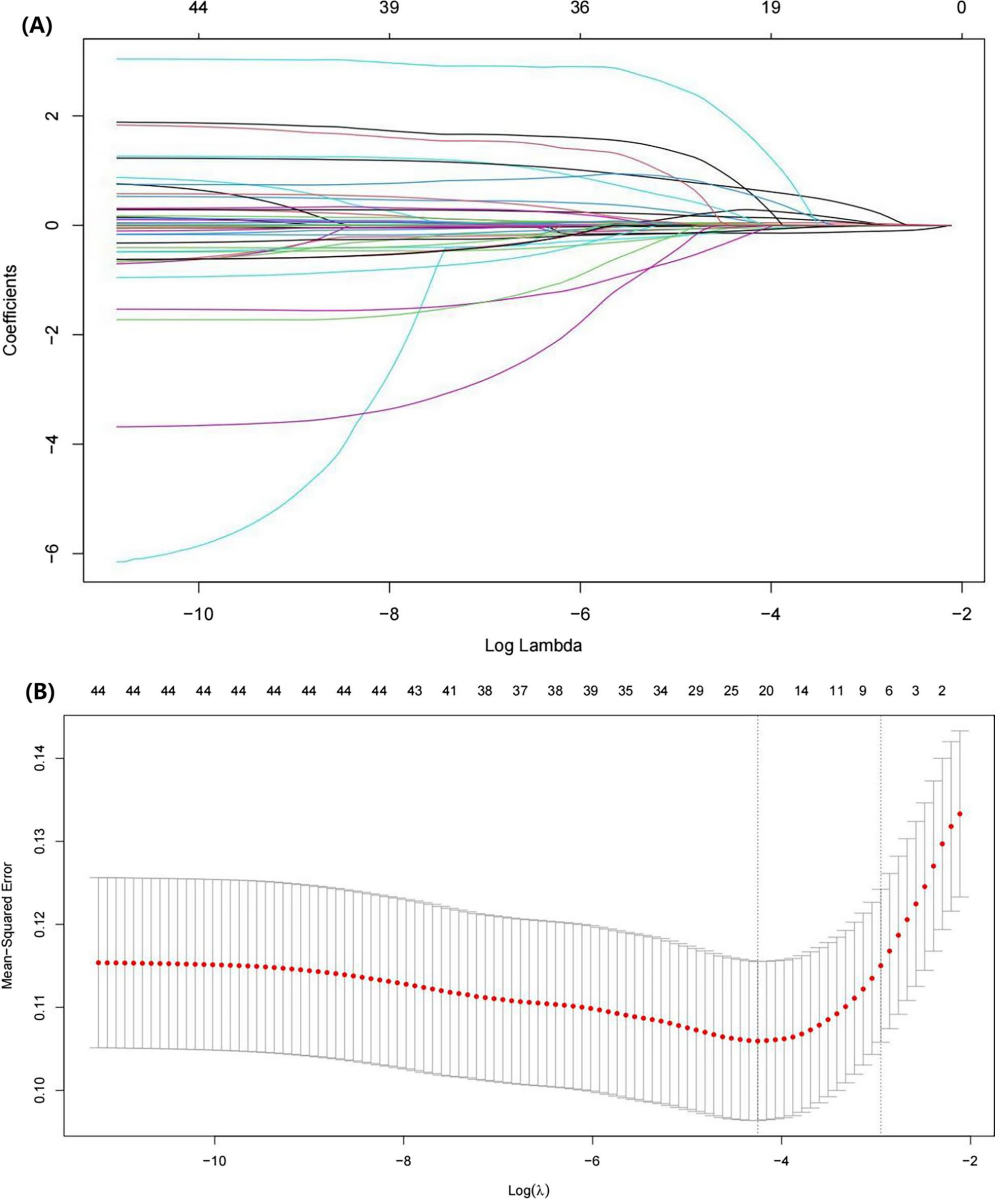
Clinical feature selection by LASSO. **A** Plot of LASSO coefficient profiles of the 44 features. The log (lambda) sequence was plotted against a coefficient profile plot. There were eight features with nonzero coefficients generated by the ideal lambda (*λ* = 0.05234919); **B** tenfold cross-validation for LASSO model parameter adjustment. The binomial deviation curve was displayed with log (lambda). The minimum criteria and its one standard error were used to construct dotted vertical lines at the optimal values (the 1-SE criteria)

**Fig. 3:**
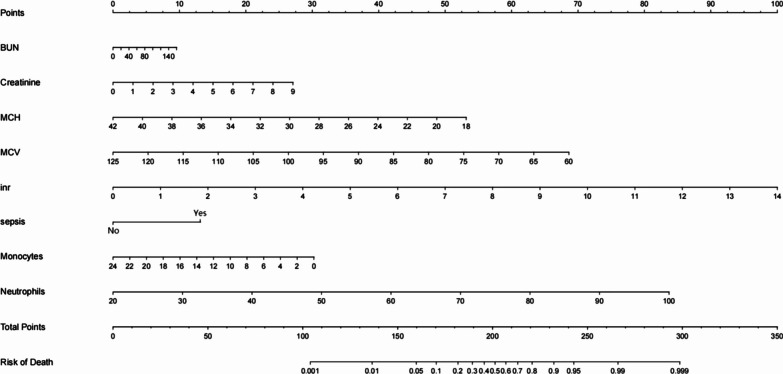
A nomogram model for predicting 30-day all-cause mortality in elderly patients with hip fracture combined with heart failure

Out of the 44 variables that were analyzed, only eight were included in the lasso logistic regression model based on the binomial bias minimum criterion (ratio 5.5:1) (Fig. 2). These variables include MCH (OR 0.85; 95% CI 0.69–1.05), MCV (OR 0.93; 95% CI 0.85–1.01), INR (OR 1.66; 95% CI 1.26–2.21), monocytes percentage (OR 0.91; 95% CI 0.77–1.07), neutrophils percentage (OR 1.07; 95% CI 1.03–1.13), BUN (OR 1.00; 95% CI 0.99–1.02), creatinine (OR 1.24; 95% CI 0.93–1.65), and combined sepsis (OR 2.54; 95% CI 1.33–4.84), where INR (*p* < 0.001), neutrophil percentage (*p* < 0.001), and combined sepsis (*p* = 0.004) were statistically significant (Table 2). Based on this, we created a nomogram that predicts mortality in elderly ICU patients with hip fracture combined with heart failure (Fig. 3).

**Table 2:** Multivariate logistic regression analysis of independent predictors of 30-day all-cause mortality in elderly patients with hip fracture combined with heart failure

Variables	OR	95%CI	*P–*value
MCH	0.855	0.692–1.051	0.13
MCV	0.928	0.854–1.006	0.07
inr	1.660	1.259–2.206	< 0.001
Monocytes	0.914	0.772–1.066	0.27
Neutrophils	1.077	1.035–1.127	< 0.001
BUN	1.004	0.988–1.021	0.61
Creatinine	1.238	0.932–1.648	0.13
Sepsis	2.537	1.330–4.838	0.004

### Apparent performance of the nomogram

**Fig. 4:**
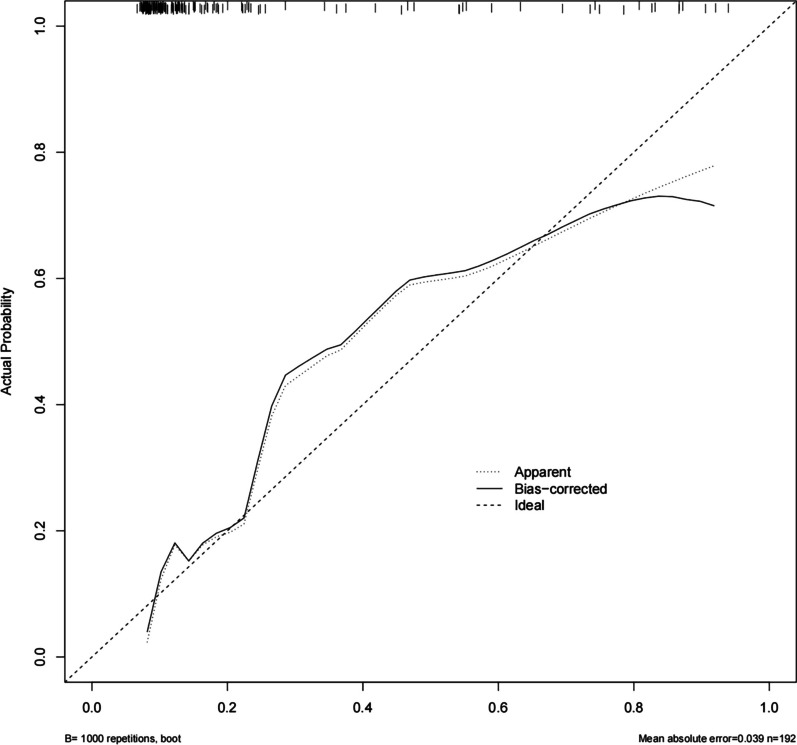
Calibration curve for patients with persistent sepsis-associated acute kidney injury is based on a nomogram

**Fig. 5:**
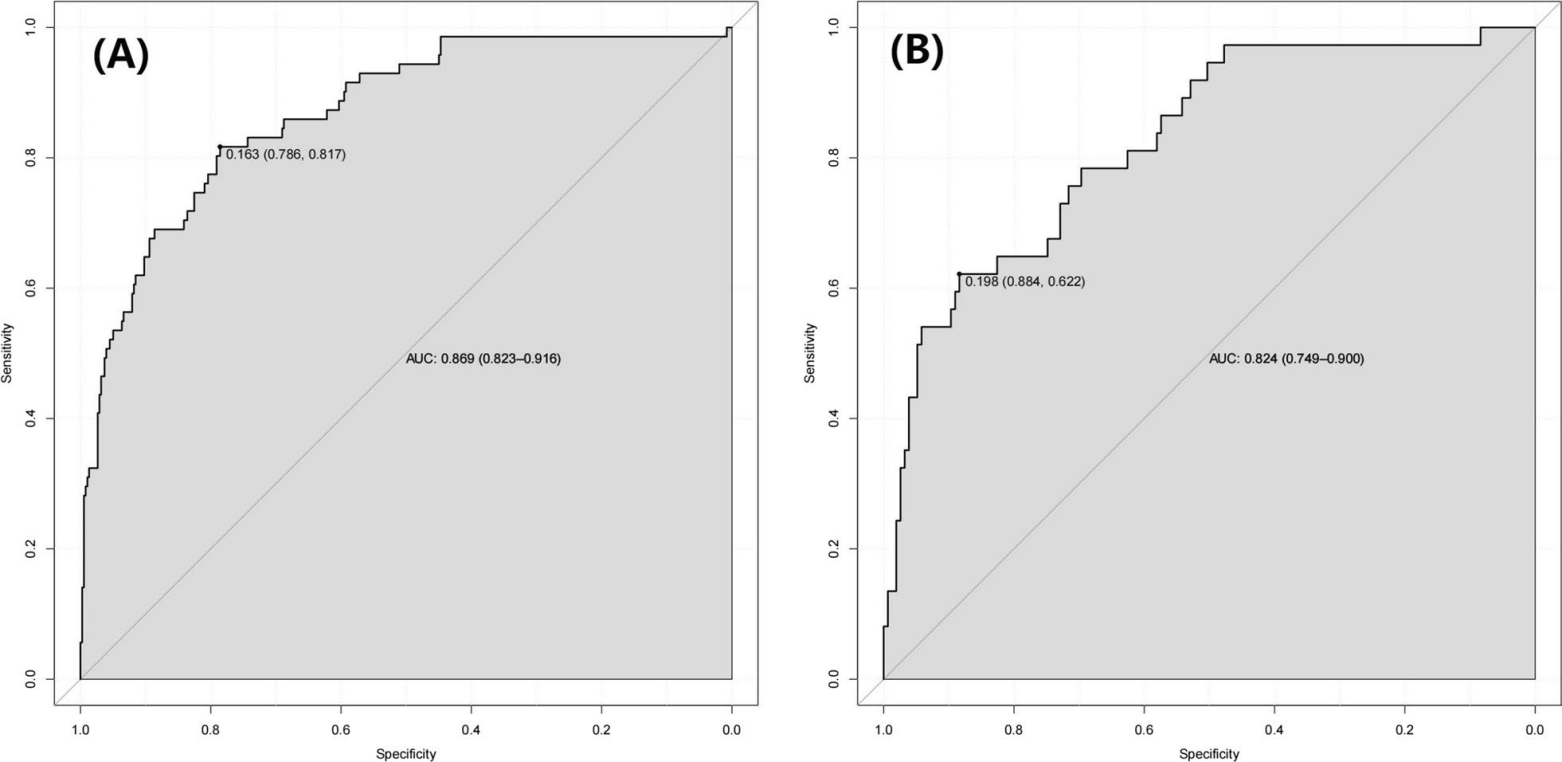
ROC of nomogram in training cohort **A** and validation cohort **B**. AUC, area under the curve; ROC, receiver operating characteristic

**Fig. 6:**
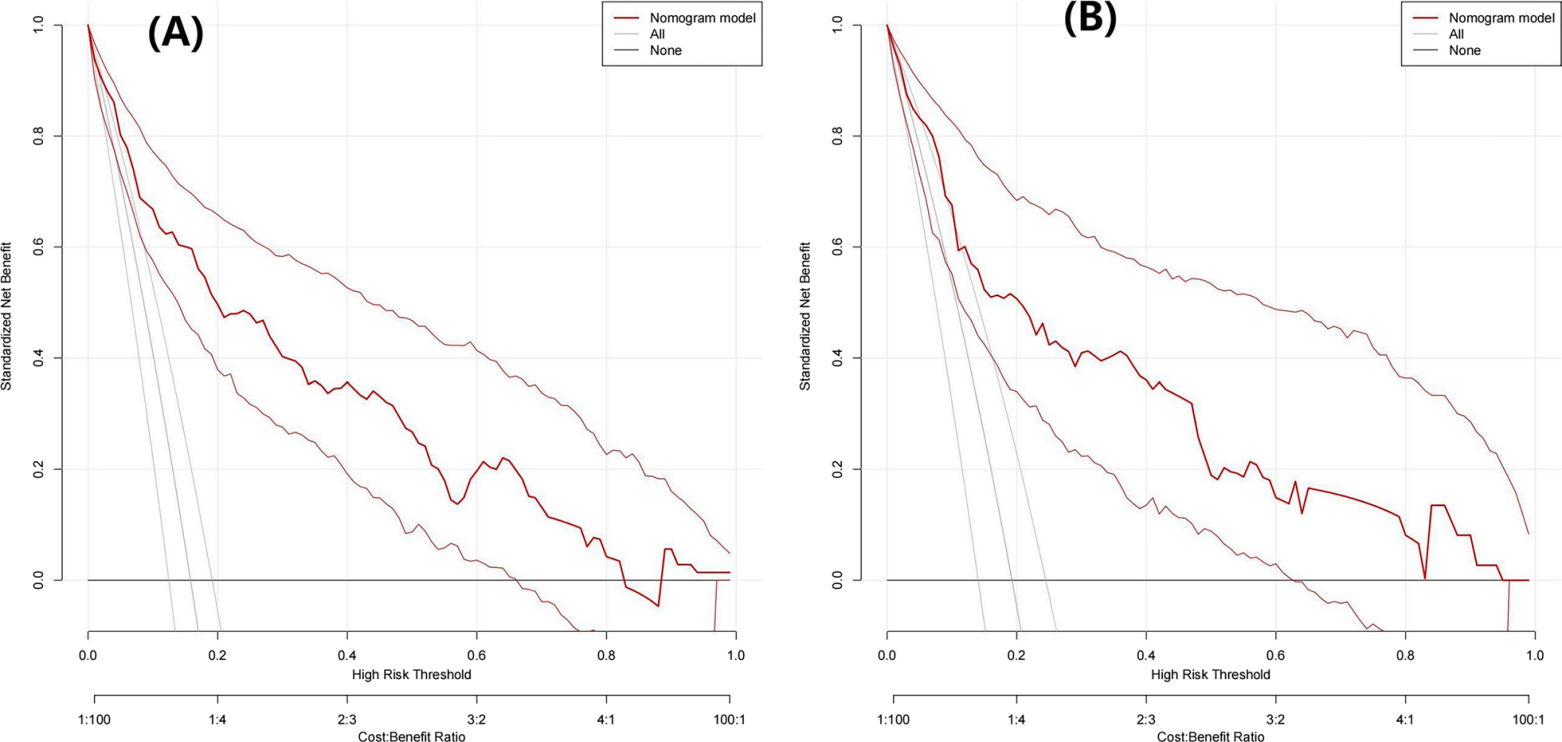
Decision curve analysis (DCA) of the nomogram model for predicting 30-day all-cause mortality in training set **A** and validation set **B**. The abscissa shows the threshold probability of 30-day all-cause mortality prediction, and the ordinate represents the net benefits of benefits and hazards. The black parallel horizontal line above the abscissa indicates that none of the patients died, and the net benefit is 0. The gray line indicates that the 30 day all-cause mortality occurred in all patients

The eight predictors that were identified were then input into R software and transformed into simple column line plots and dynamic column line plots (Fig. 3). The simple column line plots are suitable for use in clinical settings where clinicians can obtain predicted early mortality by plotting vertical lines on different score axes and summing the scores of each covariate. The nomogram plots show a good fit with the calibration curves of the training and validation sets (Fig. 4). The AUC values of the training and validation sets were 0.869 (95% CI 0.823–0.916) and 0.824 (95% CI 0.749–0.900), respectively. These results were consistent with the ROC curve analysis (Fig. 5A and B), suggesting that the nomogram model has good predictive performance for 30-day all-cause mortality. Additionally, the DCA curves demonstrated that the nomogram has a high potential clinical utility by indicating a good overall net benefit over a wide and practical range of threshold probabilities, as depicted in Fig. 6A and B.

## Discussion

Hip fractures, although common, are dangerous fractures that frequently lead to complications such as heart failure. As severe trauma weakens patients and affects their nutritional status, hip fracture patients entering the ICU are particularly susceptible to cardiopulmonary dysfunction, which can lead to acute heart failure and a higher mortality rate [14, 15]. Severe pain and reduced activity levels can also cause pulmonary infection and atelectasis, reducing oxygenation and further exacerbating cardiac load [7, 16], and triggering complications such as cardiopulmonary or neurological abnormalities with prognostic implications [17–20]. Furthermore, elderly patients often have underlying conditions concurrently such as hypertension, coronary artery disease, and heart failure, which may make them more susceptible to cardiac insufficiency. As they age, their body functions gradually weaken, their metabolic processes degenerate, and the load on their cardiovascular system increases. Therefore, in response to stimuli such as trauma, infection, and hypoxia, their cardiovascular functions are more easily and greatly impaired, leading to a significantly higher likelihood of acute deterioration of their cardiovascular system [21], and resulting in cardiac and pulmonary dysfunction, which can be complicated by acute heart failure [22]. Furthermore, systemic metabolic disorders and depletion during post-traumatic stress can further impair cardiac function and increase the likelihood of acute cardiac complications [23].

This study performed a lasso regression analysis of 641 hip fracture patients from the MIMIC-IV database to identify eight independent risk factors associated with 30-day mortality in elderly patients with hip fracture combined with heart failure in the ICU. These risk factors included monocytes percentage, neutrophils percentage, urea nitrogen, blood creatinine, MCH, MCV, and combined sepsis, all of which are common in clinical practice and are strong predictors of heart failure progression. The analysis of the developed Nomogram chart can help physicians more accurately assess the risk of acute heart failure in hip fracture patients admitted to the ICU, enabling them to develop more targeted treatment plans. This has important practical value for clinicians seeking guidance on patient management.

Both BUN and creatinine serve as indicators of kidney function, with elevated levels indicating a decrease in the screening capacity of the kidneys. Hip fracture is a significant trauma in the elderly population, and heart failure is a combination of symptoms resulting from impaired blood circulation in the pulmonary and systemic circulations. The presence of both diseases results in a simultaneous stress response, which increases the cardiovascular load of the patient and results in further impairment of renal function [24]. In a study assessing the prognosis of elderly hip fracture patients over 60 years of age, elevated BUN and creatinine were both predictive of an increased risk of short-term mortality [25]. Elevated BUN is primarily due to plasma deficit or metabolic disorders [26, while creatinine serves as a measure of renal filtration function [27]. Impaired renal function, in turn, affects the patient's cardiovascular system and increases short-term mortality [28–30. Upon admission to the ICU, patients are in critical condition and require various treatments, including extensive medications, surgery, and monitoring. While these interventions can help in restoring the patient's health, they may also have adverse effects on the kidneys and blood creatinine levels. Elevated blood creatinine often signifies compromised kidney function, which can impact essential life activities, including waste removal and fluid balance maintenance, thereby slowing down the patients' recovery [30]. Moreover, the ICU's severe recovery environment, along with therapeutic drugs and other treatments, may further impair renal function and increase blood creatinine levels. Combining the findings of this study, elevated BUN and creatinine are critical factors contributing to a heightened mortality risk within 30 days in patients with hip fracture and heart failure in the ICU. Therefore, physicians should monitor the levels of renal metabolism-related products in patients closely, reduce the drug burden, and plan treatment regimens rationally to protect renal function, slow it down, and prevent excessive renal burden from negatively affecting patients' recovery and survival, thus diminishing the risk of mortality.

Anemia is a common condition in the elderly population and is a significant factor in poor outcomes following hip fracture surgery. A study of 973 hip fracture patients found that preoperative anemia was associated with poorer physical function compared to non-anemic patients [31]. Additionally, other studies have found that hip fracture patients with anemia upon admission have a higher risk of all-cause mortality [32, 33]. Anemia and iron deficiency are also common in patients with heart failure (HF) and are associated with more severe symptoms and poor outcomes in this population [34–36]. Anemia can lead to an increased demand for oxygen by the heart, which can increase the burden on the heart and result in myocardial ischemia, myocardial injury, and even acute heart failure. Anemia may also lead to hypovolemia, causing decreased blood pressure and inadequate perfusion of blood circulation, which affects the supply of oxygen to the myocardium, exacerbating myocardial hypoxia and damage [34. The stress of operations such as surgery and anesthesia can lead to the development or worsening of anemia in patients with hip fractures, which may cause complications of acute heart failure. Moreover, patients may develop complications such as bleeding and infection during their ICU stay and undergo therapeutic measures such as diuretics, leading to higher risk of anemia and cardiovascular events [37, 38]. As MCH and MCV are indicators of anemia, this study shows that they are independent risk factors for death within 30 days in elderly patients with hip fracture and heart failure in the ICU. The underlying mechanism may result from the anemia itself and/or the chronic sequelae of the anemia [33]. However, it should be recognized that the adverse effects of allogeneic transfusions, including circulatory overload, potential infection, and allergic transfusion reactions, may put patients at higher risk for infection [39]. Systematic blood management protocols have recently been demonstrated to be effective in improving outcomes at 90 days postoperatively and should be considered for clinical use in elderly hip fracture patients with anemia who are newly admitted to the hospital [40].

Elderly patients with hip fracture combined with heart failure are a unique and high-risk group requiring ICU treatment. They often have reduced immune function, poor nutritional status, many underlying diseases, and poor cardiopulmonary function, making them vulnerable to infectious complications. Neutrophils are a type of white blood cell in the body's immune system responsible for identifying and engulfing foreign pathogens. When an infection occurs, the body's immune system is activated, resulting in an increased proportion of neutrophils; however, a continued increase in neutrophils percentage may instead have a negative impact on the body, leading to increased inflammation, tissue damage, and higher mortality in elderly patients with heart failure [41, 42]. Monocytes, responsible for removing aging and diseased cells from the body in the immune system, are reduced during sepsis development, resulting in abnormal immune responses such as a decrease in the monocytes percentage. These changes lead to a decrease in the body's immune capacity and an increase in the inflammatory response. Additionally, elderly patients with hip fracture and heart failure already have reduced immune function due to age and disease, further decreasing the monocytes percentage, leading to increased susceptibility to infection and higher mortality. Elderly patients with hip fracture combined with heart failure have reduced immune function, especially after receiving significant medication, surgery, or instrumentation, making them highly prone to infection [43]. The use of devices such as catheters and ventilators in the ICU may also increase the risk of infection, complicating sepsis and leading to increased mortality. Sepsis, an inflammatory reactive disease of the circulatory system caused by bacterial infection in the body, is a common complication for critically ill patients in the ICU, significantly affecting patient outcomes [44–46]. When combined with an increased neutrophils percentage, sepsis leads to damage to multiple organs, including the cardiovascular system, respiratory system, liver, and kidneys, among others. Acute kidney injury due to sepsis accumulation of blood creatinine and other waste products imposes an additional load on the heart, making curing heart failure patients particularly challenging and leading to higher short-term mortality [42]. Additionally, when infections occur in elderly patients with hip fracture combined with heart failure, inadequate infection management, insufficient or excessive antibiotic administration, and untimely or inadequate treatment measures can increase the risk of metastatic infection and further worsen the disease. Therefore, careful management and monitoring of infection and immune function in elderly patients with hip fracture combined with heart failure are crucial to minimize mortality risk.

In addition, in Table 2, we present the results of our multivariate logistic regression analysis concerning all-cause mortality within 30 days in elderly patients with hip fractures combined with heart failure. It is noteworthy that although we incorporated eight potential predictors into our model, the results for three of these factors were statistically significant. These three significant factors include INR (international normalized ratio), neutrophil percentage, and combined sepsis. These findings indicate a substantial influence of these three factors on predicting 30-day all-cause mortality in our study. This observation underscores the intricate nature of our model and the challenges it presents. It also underscores the importance of exercising caution when applying these results to clinical practice. Non-significant factors may warrant further investigation in larger sample sizes to gain a deeper understanding of their potential roles or associations. Despite the fact that our study was based on a combination of many ICUs across the mainland United States, there are still several limitations that need to be considered. Firstly, our data is specific to the USA, and as such, the results may not be directly applicable to ICUs in other countries. Secondly, because this was a retrospective analysis, there may be some recall bias, which suggests that a prospective cohort study would be necessary for further validation. Finally, due to the large heterogeneity of elderly patients' conditions and the complex treatment situations that occur in ICUs, we were unable to determine the causality of the independent risk factors for death. Therefore, the prediction model requires further clinical data to be evaluated and externally validated to ensure its accuracy and applicability.

## Conclusion

In this study, we identified MCH, MCV, INR, monocytes percentage, neutrophil percentage, BUN, creatinine, and combined sepsis as independent risk factors for death within 30 days in elderly patients with hip fracture combined with heart failure in the ICU. We translated the combination of these predictors into a practical columnar line graph model with strong translatability and discriminatory ability. This model can be used by clinicians to rapidly identify elderly hip fracture patients with heart failure who are at a high risk of death upon admission to the ICU. Early intervention and personalized treatment can be administered to prevent the development of potentially catastrophic outcomes. However, it is important to note that in our study, INR, neutrophil percentage, and combined sepsis demonstrated statistically significant differences. Future research can delve deeper into exploring other potential factors that may influence prognosis, providing further guidance for clinical practice and intervention strategies.

## Data Availability

Publicly available datasets were analyzed in this study. These data can be found at the datasets presented in this study can be found in online repositories. The names of the repository/repositories and accession numbers can be found at: https://physionet.org/content/mimiciv/2.0/

## Abbreviations

ICU: Intensive care unit
MIMIC-IV: Medical Information Mart for Intensive Care IV
LASSO: Least absolute shrinkage and selection operator
C-index: Consistency index
ROC: Receiver operating characteristic curve
DCA: Decision curve analysis
AUC: Area under the curve
SQL: Structured Query Language
ICD: International Classification of Diseases
MCH: Mean red blood cell hemoglobin volume
MCV: Mean red blood cell volume
RBC: Red blood cells
RDW: Red blood cell distribution width
INR: International normalized ratio
PT: Prothrombin time
PTT: Partial thromboplastin time
BUN: Blood urea nitrogen
SD: Standard deviation
IQR: Interquartile range
